# Persistent residual inflammatory risk at 1 month after contemporary PCI: rationale for routine hsCRP reassessment and dual-target therapy

**DOI:** 10.3389/fimmu.2026.1840386

**Published:** 2026-05-01

**Authors:** Xinwang Gong, Chang Zhou, Yutao Wu

**Affiliations:** 1 First Clinical Medical College, Yunnan University of Traditional Chinese Medicine, Kunming, China; 2 Special Medical Department, Dazhou Central Hospital, Dazhou, China

**Keywords:** hsCRP, NLRP3 inflammasome, percutaneous coronary intervention, precision anti-inflammatory therapy, residual inflammatory risk

## Abstract

Despite major advances in lipid-lowering therapy and stent technology, a substantial proportion of patients undergoing contemporary percutaneous coronary intervention (PCI) experience recurrent major adverse cardiovascular events (MACE) driven by persistent residual inflammatory risk (RIR). This Perspective highlights that, once LDL−C is optimized below 70 mg/dL, RIR—quantified by high−sensitivity C−reactive protein (hsCRP ≥2 mg/L)—emerges as the dominant modifiable driver of recurrent events. Landmark meta−analyses and large registries demonstrate that persistent elevation of hsCRP at 1 month post−PCI occurs in approximately 43% of patients and independently predicts 12−month MACE (RR 1.64), all−cause mortality (RR 3.25), and other adverse outcomes, outperforming residual cholesterol risk after multivariable adjustment. Mechanistically, the acute−phase hsCRP surge induced by procedural injury resolves by 4–6 weeks, after which the 1−month value reliably reflects ongoing NLRP3 inflammasome activation, IL−1β/IL−6 signaling, and macrophage−driven plaque inflammation rather than transient artefacts. Accordingly, we propose a new “dual−target” definition of optimal secondary prevention: achievement of both LDL−C <70 mg/dL and hsCRP <2 mg/L at the 1−month landmark. This biomarker−guided approach represents a hypothesis-generating framework to enable precision deployment of low−dose colchicine or IL−6 pathway inhibitors (e.g., ziltivekimab) in patients with persistent RIR, directly addressing the enrichment gap observed in neutral broad anti−inflammatory trials such as CLEAR−SYNERGY. We therefore propose consideration of routine 1−month hsCRP reassessment as a potential future Class IIa recommendation in ESC/ACC guidelines. This strategy may transform silent residual inflammatory risk into a precisely treatable immunologic target, pending prospective validation in dedicated trials.

## Introduction

Percutaneous coronary intervention (PCI) has advanced dramatically with second- and third-generation drug-eluting stents, routine intravascular imaging (IVUS or OCT), and aggressive lipid-lowering regimens combining high-intensity statins with ezetimibe or PCSK9 inhibitors. These strategies routinely achieve LDL-C levels below 70 mg/dL in most patients. Nevertheless, real-world registries continue to report recurrent major adverse cardiovascular events (MACE) in approximately 10–15% of patients within the first year after contemporary PCI, with substantially higher rates in certain high-risk subsets (1). This persistent vulnerability highlights that coronary atherosclerosis is not solely a lipid-driven disease but also an immuno-inflammatory condition in which vascular-wall inflammation and plaque microenvironmental activity continue unabated even after optimal lipid control (2).

Once LDL-C is successfully lowered, residual inflammatory risk (RIR) emerges as the dominant unmet therapeutic target. RIR is most practically and reliably quantified by high-sensitivity C-reactive protein (hsCRP), a downstream marker of NLRP3 inflammasome activation, IL-1β/IL-6 signalling, and macrophage-driven plaque inflammation. A landmark systematic review and meta-analysis published in 2026 by Romeo and colleagues pooled five contemporary PCI cohorts totalling 13,604 patients with paired hsCRP measurements (baseline and 1 month ±5 days post-procedure) (3). High RIR was defined as hsCRP ≥2 mg/L at the 1-month follow-up—a threshold identical to that used in the CANTOS trial. Strikingly, 5,833 patients (42.9%) still exhibited persistent high RIR despite modern secondary prevention. At 12-month follow-up, this persistent elevation was associated with a 1.64-fold increased risk of MACE (random-effects risk ratio [RR] 1.64, 95% CI 1.33–2.03; I²=80.2%), a 3.25-fold increase in all-cause mortality (RR 3.25, 95% CI 2.49–4.25; I²=67%), a 1.46-fold higher risk of non-fatal myocardial infarction (RR 1.46, 95% CI 1.00–2.12), and a 1.64-fold increase in non-fatal stroke (RR 1.64, 95% CI 1.14–2.37). Sensitivity analyses, including leave-one-out and Baujat plots, confirmed that the direction and magnitude of these associations remained robust even after accounting for heterogeneity. The findings were consistent across Western and Asian populations, underscoring the universal relevance of persistent RIR.

Complementing this meta-analytic evidence, a large single-centre registry by Bay and colleagues (2025) analysed 15,494 statin-treated patients undergoing PCI between 2012 and 2022 (4). Patients were stratified at 1 month into four groups according to LDL-C (<70 vs ≥70 mg/dL) and hsCRP (<2 vs ≥2 mg/L). Isolated residual inflammatory risk conferred the highest 1-year MACE rate (5.1%), exceedingly even the combined residual risk group. After multivariable adjustment, isolated RIR carried an adjusted hazard ratio (aHR) of 1.78 (95% CI 1.36–2.33), while isolated residual cholesterol risk showed no independent association (aHR 1.01, 95% CI 0.76–1.35). These data unequivocally demonstrate that, once LDL-C is optimised, inflammation—not cholesterol—drives the majority of residual events in contemporary practice.

Despite this mechanistic framework, current ESC and ACC guidelines recommend only baseline hsCRP assessment in selected high-risk patients and do not mandate routine repeat measurement at the 1-month post-PCI visit (5–7). This policy gap is critical: serial measurements in post-PCI cohorts demonstrate that the acute-phase hsCRP surge induced by myocardial injury and stent implantation typically peaks within the first 24–48 hours and substantially subsides by 4–6 weeks (8, 9), at which point the 1-month value reliably reflects chronic plaque and perivascular inflammation rather than procedural artefacts. Without systematic 1-month reassessment, clinicians remain unable to identify the substantial subset of patients (over 40%) who would derive the greatest absolute benefit from targeted anti-inflammatory therapies. The present Perspective therefore proposes as a hypothesis-generating framework that routine 1−month hsCRP reassessment may enable precision deployment of colchicine or IL−6 pathway inhibitors, directly connecting mechanistic insights to clinically actionable patient stratification. Prospective, biomarker−guided randomized trials are urgently needed to validate this strategy.

## Current evidence

### Evidence 1: 1-month hsCRP predicts outcomes irrespective of disease acuity

Song and colleagues (9) prospectively enrolled 4,263 East Asian patients undergoing PCI (2,376 with acute myocardial infarction, 1,887 with stable disease) to evaluate the comparative temporal profiles of hsCRP and their prognostic implications. During the first month post-PCI, elevated baseline hsCRP predicted early MACE only in AMI patients. In contrast, during the late phase (1 month to 4 years), a 1-month hsCRP ≥1.6 mg/L predicted MACE irrespective of presentation, with an adjusted HR of 2.40 in AMI and 2.67 in stable CAD patients. This finding is critical: it demonstrates that the 1-month measurement captures chronic inflammatory burden that transcends the acute-phase variability associated with myocardial infarction, supporting its validity as a stable discriminator of residual inflammatory risk regardless of presentation.

### Evidence 2: prognostic dominance of 1-month hsCRP across contemporary cohorts

Cho and colleagues (8) performed a comprehensive biomarker assessment in 2,789 post-PCI patients, demonstrating a clear dose–response relationship between 1-month hsCRP quartiles and 4-year MACE risk, with 1-month hsCRP showing the strongest prognostic value among all biomarkers evaluated. Similarly, Yang et al. (10) confirmed in 2,376 Chinese patients that 1-month hsCRP significantly predicted 12-month MACCE, whereas baseline hsCRP did not. Collectively, these studies establish that the 1-month measurement—rather than baseline values—provides the most reliable prognostic information for guiding chronic secondary prevention.

### Evidence 3: prevalence and prognostic impact of persistent RIR (Romeo 2026 meta-analysis)

Romeo et al. (3) performed the first dedicated systematic review and meta-analysis focused exclusively on serial hsCRP in the contemporary PCI era. Five studies met inclusion criteria, all requiring paired measurements at baseline and >4 weeks post-PCI. The pooled prevalence of persistent high RIR (hsCRP ≥2 mg/L at 1 month) reached 42.9%. The primary endpoint analysis demonstrated a statistically significant and clinically meaningful increase in 12-month MACE (RR 1.64). Secondary endpoints revealed even stronger associations with all-cause mortality (RR 3.25) and stroke (RR 1.64), with moderate heterogeneity for mortality (I²=67%) largely attributable to one outlier study. Exclusion of this study reduced heterogeneity substantially without altering the overall conclusions. Subgroup analyses confirmed consistency across continents, stent generations, and clinical presentations (stable CAD vs acute coronary syndromes). The authors explicitly concluded that serial hsCRP assessment post-PCI could guide both intensified lipid-lowering and anti-inflammatory strategies, highlighting the need for further prospective validation.

### Evidence 4: isolated RIR outperforms residual cholesterol risk (Bay 2025 cohort)

Bay and colleagues (4) leveraged one of the largest contemporary PCI registries to dissect the relative contributions of residual cholesterol versus inflammatory risk. Among 15,494 statin-treated patients, four distinct risk phenotypes were defined at the 1-month landmark. Isolated RIR (LDL-C <70 mg/dL but hsCRP ≥2 mg/L) was associated with the highest crude MACE incidence (5.1%) and retained the strongest independent prognostic signal after extensive multivariable adjustment (aHR 1.78). In contrast, isolated residual cholesterol risk (LDL-C ≥70 mg/dL but hsCRP <2 mg/L) conferred no additional hazard. These findings align with earlier collaborative analyses of statin-treated populations showing that hsCRP is a more potent predictor of recurrent events than LDL-C once intensive lipid-lowering is achieved (11). The study further demonstrated that patients with combined residual risk experienced intermediate hazard (aHR 1.56), reinforcing the primacy of inflammation in the modern era.

### Evidence 5: why broad anti-inflammatory trials have been neutral and how patient selection matters

The CLEAR-SYNERGY (OASIS 9) trial (12, 13) randomised 7,062 patients with acute myocardial infarction undergoing PCI to low-dose colchicine (0.5 mg daily) or placebo. Despite a statistically significant reduction in CRP levels, the primary composite endpoint (cardiovascular death, recurrent MI, stroke, or ischemia-driven revascularisation) was neutral (HR 0.99, 95% CI 0.85–1.16) at 3-year median follow-up. In contrast, COLCOT and LoDoCo2 demonstrated clear benefit in populations enriched for higher baseline inflammatory burden or stable disease (14, 15). Similarly, the CANTOS trial with canakinumab showed that clinical benefit was confined to participants achieving on-treatment hsCRP <2 mg/L (16). More recent biomarker-focused cohorts, such as Cho et al. (8), confirmed that only 1-month hsCRP ≥2 mg/L and fibrinogen independently predicted 3-year all-cause mortality after ACS, whereas LDL-C and HbA1c did not. The consistent lesson across these studies is that unselected initiation of anti-inflammatory therapy—particularly when initiated during the acute phase without regard to post-stabilisation inflammatory burden—dilutes efficacy. While factors such as timing of initiation and potential drug–drug interactions may have contributed to the neutral result of CLEAR-SYNERGY, the most critical insight is that enrichment based on persistent hsCRP ≥2 mg/L at the 1-month window represents a promising, yet unproven, strategy that may replicate and potentially exceed the benefits observed in landmark trials. A summary of the key contemporary evidence supporting the prognostic superiority and clinical utility of 1-month hsCRP assessment is provided in **Table 1**. These data collectively underscore that persistent residual inflammatory risk at 1 month, rather than baseline values or residual cholesterol risk, represents the dominant and potentially modifiable driver of recurrent events in the modern PCI era.

**Table 1:** Key contemporary evidence supporting routine 1-month hsCRP assessment after PCI.

Study/evidence source	Design & population	Key finding	Hazard ratio/risk ratio (95% CI)	Clinical implication
Romeo et al. (2026) (3)	Meta-analysis (5 cohorts, n=13,604)	Persistent high RIR (hsCRP ≥2 mg/L at 1 month) in 42.9% of patients	MACE RR 1.64 (1.33–2.03); All-cause mortality RR 3.25 (2.49–4.25)	Strong observational evidence for serial hsCRP to guide risk stratification
Bay et al. (2025) (4)	Large registry (n=15,494 statin-treated PCI patients)	Isolated RIR had highest 1-year MACE rate (5.1%); isolated cholesterol risk neutral	Isolated RIR aHR 1.78 (1.36–2.33)	Inflammation drives residual risk more than LDL-C in modern era
Song et al. (2023) (9)	Prospective cohort (n=4,263 East Asian PCI patients)	1-month hsCRP ≥1.6 mg/L predicts late MACE irrespective of ACS vs stable CAD	AMI aHR 2.40; Stable CAD aHR 2.67	1-month value captures chronic inflammation beyond acute-phase response
Cho et al. (2026) (8)	Biomarker cohort (n=2,789 post-PCI)	Dose-response between 1-month hsCRP quartiles and 4-year MACE; strongest prognostic marker	Clear dose-response (exact HR not pooled in text)	1-month hsCRP outperforms baseline and other biomarkers
CLEAR-SYNERGY vs COLCOT/LoDoCo2/CANTOS (12–16)	RCTs	Neutral result in unselected acute PCI; benefit in enriched/inflamed or stable populations	CLEAR-SYNERGY HR 0.99 (neutral); Benefit confined to on-treatment hsCRP <2 mg/L in CANTOS	Patient selection by persistent 1-month hsCRP is a hypothesis for future trials

RIR, residual inflammatory risk; MACE, major adverse cardiovascular events; aHR, adjusted hazard ratio; RR, risk ratio; ACS, acute coronary syndrome; CAD, coronary artery disease. All hsCRP thresholds are ≥2 mg/L unless otherwise specified. Data reflect contemporary PCI with modern stents and lipid-lowering therapy.

## Why 1 month is the critical window and mechanistic gap

hsCRP exhibits marked fluctuation in the first 2–4 weeks after PCI due to procedural myocardial injury, stent-induced endothelial activation, and acute-phase response. By 4–6 weeks, levels stabilise and accurately mirror ongoing NLRP3 inflammasome activity, macrophage infiltration, and perivascular adipose tissue inflammation within the coronary plaque microenvironment (8, 17). For patients presenting with acute coronary syndromes, the baseline hsCRP value predominantly captures the acute-phase response to myocardial injury and retains prognostic value for very early (30-day) events; for those undergoing elective PCI for stable coronary artery disease, baseline levels may partially reflect chronic inflammation. In both scenarios, however, for the purpose of guiding chronic secondary prevention beyond the periprocedural period, the 1-month value provides a more accurate representation of post-procedural residual plaque inflammation that is both biologically meaningful and clinically actionable. The 1-month time point is logistically ideal, coinciding with the first routine outpatient visit and requiring only a standard blood draw.

Although circulating IL-6 is more proximal to the causal inflammatory cascade, hsCRP remains the preferred bedside marker because of its widespread availability, standardisation, low cost, and proven responsiveness to both colchicine (NLRP3 inhibition) and IL-6 ligand inhibitors such as ziltivekimab. IL-6 ligand blockade suppresses upstream inflammatory signalling, leading to downstream reduction in hsCRP; thus, hsCRP serves as an ideal pharmacodynamic marker for patient selection and treatment monitoring (18). The NLRP3 inflammasome represents the molecular hub linking metabolic stress to sustained vascular inflammation: priming via TLR activation induces NLRP3 and pro-IL-1β transcription, while a second signal—such as cholesterol crystals, mitochondrial ROS, or potassium efflux—triggers caspase-1 activation, IL-1β/IL-6 production, and pyroptosis, perpetuating plaque vulnerability (19, 20).

**Figure 1:**
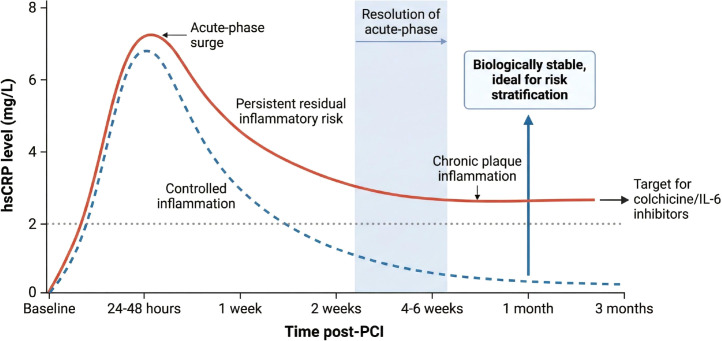
Schematic representation of the typical hsCRP kinetic profile after contemporary PCI and the rationale for 1-month reassessment. This figure is a schematic (illustrative) representation adapted from serial hsCRP measurements reported in contemporary PCI cohorts (8, 9, 17). It is not derived from a single primary empirical dataset. hsCRP levels typically peak within 24–48 hours due to procedural myocardial injury and stent implantation, then decline. By 4–6 weeks, values stabilize and reflect ongoing NLRP3 inflammasome activity and chronic plaque inflammation rather than acute artefacts. The 1-month time point (vertical dashed line) is both biologically meaningful and logistically ideal, coinciding with the first routine outpatient visit. Persistent elevation ≥2 mg/L identifies patients who may derive greatest benefit from targeted anti-inflammatory therapy.

We therefore propose a new “dual-target” definition of optimal secondary prevention after contemporary PCI as a conceptual framework: achievement of both LDL-C <70 mg/dL and hsCRP <2 mg/L at the 1-month landmark. This approach is supported by the IMPROVE-IT trial analysis (21), in which patients achieving dual targets at 1 month had a 27% relative risk reduction for major ASCVD events compared with those achieving neither target. Patients failing the hsCRP target represent a mechanistically distinct endotype characterised by persistent NLRP3-driven plaque vulnerability and could be prioritized for investigation of intensified anti-inflammatory interventions in future clinical trials. This approach directly bridges vascular biology (plaque microenvironmental inflammation) with pragmatic, biomarker-guided risk stratification. Importantly, hsCRP is not only a prognostic biomarker but also a dynamic pharmacodynamic marker responsive to both NLRP3 inhibition (colchicine) and direct IL-6 blockade (ziltivekimab), enabling future treat-to-target strategies (22). **Figure 1** illustrates the typical hsCRP kinetic profile after contemporary PCI and highlights the rationale for 1-month reassessment. As shown, hsCRP levels typically peak within 24–48 hours due to procedural myocardial injury and stent implantation, then decline; by 4–6 weeks, values stabilize and reflect ongoing NLRP3 inflammasome activity and chronic plaque inflammation rather than acute artefacts, making the 1-month time point both biologically meaningful and logistically ideal.

## Limitations of the current evidence

Despite the compelling associations, several limitations warrant caution. First, hsCRP is a nonspecific acute-phase reactant that can be elevated by non-cardiovascular conditions such as infection, obesity, or chronic inflammatory comorbidities, potentially leading to false-positive identification of RIR. Second, the Romeo et al. meta-analysis demonstrated substantial statistical heterogeneity (I²=80.2% for MACE), with sensitivity analyses indicating moderate influence from individual studies; although directionality remained consistent, this reduces certainty in the pooled estimates. Third, publication bias cannot be entirely excluded in observational meta-analyses, despite the use of Baujat plots. Finally, and most importantly, the proposed biomarker-guided anti-inflammatory strategy, while mechanistically sound and supported by observational data, lacks validation in large-scale prospective randomized trials specifically enriched by 1-month hsCRP. These caveats underscore that our dual-target proposal remains hypothesis-generating rather than immediately practice-changing.

## Clinical and policy recommendations

Implementation for risk stratification is straightforward and low-cost. Every PCI patient could undergo hsCRP measurement before discharge and again at the scheduled 1-month follow-up visit. This low-cost step identifies the 40%+ subset with persistent RIR without requiring additional infrastructure or advanced imaging.

While the following tiered algorithm is conceptual and hypothesis-generating, it is grounded in the mechanistic and observational data summarized above:

• Universal step: Measure hsCRP before discharge and at the 1-month outpatient visit in all PCI patients with optimized LDL-C <70 mg/dL.
• If persistent RIR (hsCRP ≥2 mg/L at the 1−month landmark) and stable renal/hepatic function: Pending results of ongoing randomized trials, initiation of low-dose colchicine 0.5 mg daily could be considered as a potential strategy to address the enrichment gap observed in the neutral CLEAR−SYNERGY trial; the ongoing COL BE PCI trial (NCT06095765) (23, 24) is specifically testing this post-PCI strategy in patients selected on the basis of post−stabilisation inflammatory burden and will provide definitive safety and efficacy data.
• In higher-risk individuals (diabetes, multivessel disease, prior events) or those intolerant to colchicine: IL-6 ligand inhibition with ziltivekimab might be evaluated within ongoing trial frameworks (e.g., ARTEMIS trial, NCT06118281) (25) as an upstream anti−inflammatory strategy targeting the NLRP3–IL−6 axis. The results of ARTEMIS are expected to further refine the role of IL−6 pathway inhibition in hsCRP−guided precision therapy.

Guideline committees may wish to consider incorporating 1-month hsCRP reassessment as a potential Class IIa recommendation in future ESC/ACC updates, pending confirmatory evidence from ongoing trials, and endorse the dual-target (LDL-C + hsCRP) endpoint as a framework for secondary prevention research. Future randomised trials would benefit from adopting hsCRP-guided enrichment at the 1-month landmark—mirroring the success of CANTOS and avoiding the dilution observed in CLEAR-SYNERGY. Safety monitoring remains essential: gastrointestinal events with colchicine and infection risk with biologics are manageable with routine clinical oversight. Artificial intelligence-assisted reporting of hsCRP trends within electronic health records could further streamline implementation and longitudinal monitoring.

These recommendations align precisely with the scope of the present Research Topic by integrating mechanistic insights into plaque inflammation, biomarker-enabled risk stratification, and precision therapeutic strategies targeting residual inflammatory pathways.

## Conclusion

A single, inexpensive hsCRP measurement performed 1 month after contemporary PCI can unmask persistent residual inflammatory risk in more than 40% of patients and may identify those who stand to gain the greatest absolute benefit from colchicine or emerging IL-6 inhibitors. Routine reassessment at discharge and 1 month represents a promising strategy to transform RIR from a silent driver of recurrent events into a precisely treatable target. Consideration of this simple step in future guidelines, combined with hsCRP-guided trial enrichment, could potentially reduce MACE and mortality while accelerating the next generation of inflammation-targeted therapies. The observational evidence is compelling; however, definitive prospective validation in randomized trials is required before this approach can be considered standard of care. One blood test at the 1-month visit offers a potential pathway to meaningfully improve outcomes for tens of thousands of PCI patients worldwide each year.

## Data Availability

The original contributions presented in the study are included in the article/supplementary material. Further inquiries can be directed to the corresponding author.
